# Neurofilament light chain and α-synuclein RT-QuIC as differential diagnostic biomarkers in parkinsonisms and related syndromes

**DOI:** 10.1038/s41531-021-00232-4

**Published:** 2021-10-11

**Authors:** Corinne Quadalti, Giovanna Calandra-Buonaura, Simone Baiardi, Andrea Mastrangelo, Marcello Rossi, Corrado Zenesini, Giulia Giannini, Niccolò Candelise, Luisa Sambati, Barbara Polischi, Giuseppe Plazzi, Sabina Capellari, Pietro Cortelli, Piero Parchi

**Affiliations:** 1 IRCCS, Istituto delle Scienze Neurologiche di Bologna, Bologna, Italy; 2 Department of Biomedical and Neuromotor Sciences (DIBINEM), University of Bologna, Bologna, Italy; 3 Department of Experimental, Diagnostic and Specialty Medicine (DIMES), University of Bologna, Bologna, Italy; 4 Department of Biomedical, Metabolic and Neural Sciences, University of Modena and Reggio Emilia, Modena, Italy

**Keywords:** Parkinson's disease, Diagnostic markers

## Abstract

Neurofilament light chain (NfL) and α-synuclein oligomeric seeds (α-syn-s) are promising biomarkers for patients with parkinsonism. We assessed their performance in discriminating Parkinson disease (PD) from atypical parkinsonisms (APDs) and evaluated the association between NfL levels and clinical measures of disease severity. We measured NfL in cerebrospinal fluid (CSF) and/or plasma by immunoassays and α-syn-s in CSF by real-time quaking-induced conversion (RT-QuIC) in patients with PD (*n* = 153), multiple system atrophy (MSA) (*n* = 80), progressive supranuclear palsy/cortico-basal syndrome (PSP/CBS) (*n* = 58), dementia with Lewy bodies (*n* = 64), isolated REM-sleep behaviour disorder (*n* = 19), and isolated autonomic failure (*n* = 30). Measures of disease severity included disease duration, UPDRS-III score, Hoehn and Yahr stage, orthostatic hypotension, MMSE score, and CSF amyloid-beta profile. Both CSF NfL (cNfL) and plasma NfL (pNfL) levels were markedly elevated in APDs, and allowed differentiation with PD (vs. APDs, cNfL AUC 0.96; pNfL AUC 0.95; vs. MSA cNfL AUC 0.99; pNfL AUC 0.97; vs. PSP/CBS cNfL AUC 0.94; pNfL AUC 0.94). RT-QuIC detected α-syn-s in 91.4% of PD, but only 2.5% of APDs (all MSA). In PD/PDD, motor scales significantly correlated with cNfL levels. Although pNfL and both cNfL and α-syn-s accurately distinguished PD from APDs, the combined assessment of CSF markers provided a higher diagnostic value (PD vs. APDs AUC 0.97; vs. MSA AUC 0.97; vs. PSP/CBS AUC 0.99) than RT-QuIC alone (*p* = 0.047 vs. APDs; *p* = 0.002 vs MSA; *p* = 0.007 vs PSP/CBS), or cNfL alone (*p* = 0.011 vs. APDs; *p* = 0.751 vs MSA; *p* = 0.0001 vs. PSP/CBS). The results support the use of these assays in specialised clinics.

## Introduction

Parkinson disease (PD) is clinically difficult to discriminate from atypical parkinsonism disorders (APDs), namely multiple system atrophy (MSA), progressive supranuclear palsy (PSP), and cortical basal syndrome (CBS), especially during the early disease stages. Current diagnostic criteria for both PD and APDs have some limitations because they require a combination of clinical findings, multiple diagnostic investigations, and an adequate follow-up of several years to reach an accurate disease identification^1,2^. The growing interest towards early and accurate identification of neurodegenerative diseases makes the search for in vivo molecular markers of diagnostic and prognostic value for PD and APDs of fundamental importance^3^.

In recent years, two novel biomarkers have received much attention in this field. Neurofilament light chain (NfL), a protein marker of neuro-axonal degeneration, has been reported to be of value in several neurologic conditions, including PD and APDs, that are associated with rapid disease progression or considerable neuronal damage in subcortical regions^4,5^. Specifically, NfL concentration in both cerebrospinal fluid (CSF) and blood is significantly higher in patients with APDs than in patients with PD and, therefore, of value in distinguishing the two disease groups^6–10^. Even higher interest, given their specificity, has been raised by novel ultrasensitive in vitro assays exploiting the template prion-like capacities of the pathogenic protein as an amplification strategy to detect a minute amount of disease-specific misfolded, aggregated forms of α-synuclein (α-syn) in CSF^11^. We and others have recently shown that abnormal α-syn aggregates can be reliably detected in the CSF of patients with PD by real-time quaking-induced conversion (RT-QuIC), allowing a differentiation between APDs and PD due to Lewy body disease (LBD)^12–16^.

In this study, we sought to further explore the value of NfL and α-syn seeding activity in biofluids, either alone or in combination, as diagnostic and prognostic biomarkers in a large series of patients presenting with parkinsonism and related syndromes, including PD, MSA, PSP, CBS, dementia with Lewy bodies (DLB), PD dementia (PDD), isolated autonomic failure (iAF) and isolated REM sleep behavior disorder (iRBD).

## Results

### Demographic variables and comparison of the diagnostic value of plasma and CSF NfL levels in the differential diagnosis of patients with parkinsonism

Among patients with parkinsonism, those from the PD and MSA groups were significantly younger than patients with PSP/CBS, PDD, and DLB, and sex distribution was characterised by a higher prevalence of males in all patient groups, except for MSA (Table 1). Moreover, there were no differences in time from clinical onset to sample collection between the patient groups, except for PD versus DLB or PDD. Additional demographic differences for patients with prodromal syndromes (iRBD and iAF) and controls are shown in Table 1.

**Table 1:** Demographic data of the study cohort.

	PD	PDD	DLB	MSA	PSP/CBS	iAF	iRBD	Controls
	(*n* = 116)	(*n* = 37)	(*n* = 64)	(*n* = 80)	(*n* = 58)	(*n* = 30)	(*n* = 19)	(*n* = 72)
Age (yrs)	59.9 ± 10.3^a-e^	69.6 ± 8.1 ^f,g^	73.8 ± 5.7 ^f-h^	61.1 ± 8.0^c^	71.2 ± 6.8 ^g^	65.5 ± 7.9^i^	67.3 ± 7.4 ^g^	58.1 ± 10.1
Female (%)	30.2^k–m^	16.2^i,m,n^	29.7^k,o^	53.8	46.5	33.3	31.6	52.8
UPDRS-III	16.3 ± 8.5^b,c,f^	27.1 ± 18.1	33.9 ± 11.2	33.6 ± 11.9	38.9 ± 25.4	NA	NA	NA
Hoehn & Yahr	1.5 ± 0.6^c,f,p,q^	2.3 ± 0.9^r^	2.1 ± 0.7 ^m^	3.1 ± 2.9	2.7 ± 0.8	NA	NA	NA
MMSE	28.9 ± 1.8^a-c^	24.0 ± 4.4 ^f,s^	23.3 ± 5.2^d,f,s^	28.1 ± 2.0 ^l^	25.1 ± 5.4^t^	27.9 ± 1.9	29.2 ± 1.2	NA
Onset to collection (mos)	46.2 ± 51.8^q,u,v^	73.9 ± 52.6	75.9 ± 90.9^d^	51.2 ± 31.9 ^h^	50.8 ± 31.2^w^	101.9 ± 54.9	71.0 ± 69.7	NA

Data are expressed as mean ± SD. Depending on data distribution, demographic and clinical features were compared using chi-square and one-way ANOVA (followed by Bonferroni’s post-hoc analysis) or Kruskal–Wallis (followed by Dunn’s post-hoc analysis) tests: ^a^compared to PDD ≤ 0.0001. ^b^compared to DLB ≤ 0.0001. ^c^compared to PSP/CBS ≤ 0.0001. ^d^compared to iAF ≤ 0.05. ^e^compared to iRBD ≤ 0.05. ^f^compared to MSA ≤ 0.0001. ^g^compared to controls ≤ 0.0001. ^h^compared to iAF ≤ 0.001. ^I^compared to controls ≤ 0.001. ^k^compared to controls ≤ 0.01. ^l^compared to PSP/CBS ≤ 0.05. ^m^compared to MSA ≤ 0.001. ^n^compared to PSP/CBS ≤ 0.01. ^o^compared to MSA ≤ 0.01. ^p^compared to PDD ≤ 0.01. ^q^compared to DLB ≤ 0.01. ^r^compared to MSA ≤ 0.05. ^s^compared to iRBD ≤ 0.0001. ^t^compared to iRBD ≤ 0.01. ^u^compared to PDD ≤ 0.001. ^v^compared to iAF ≤ 0.0001. ^w^compared to iAF ≤ 0.01.

*UPDRS* Unified Parkinson’s Disease Rating Scale, *MMSE* Mini-Mental State examination, *NA* not applicable.

Age was associated with pNfL levels in patients with PD (rho = 0.49, *p* < 0.0001) and PSP/CBS (rho = 0.44, *p* = 0.005) and controls (rho = 0.71, *p* < 0.0001), but not in patients with MSA. Similarly, age was associated with cNfL levels in patients with PD (rho = 0.50, *p* < 0.0001) and controls (rho = 0.63, *p* = 0.005), but not in patients with PSP/CBS or MSA. In contrast, sex showed no effect on blood and CSF biomarker values. Accordingly, all comparisons of pNfL and cNfL between groups were adjusted for age.

**Fig. 1:**
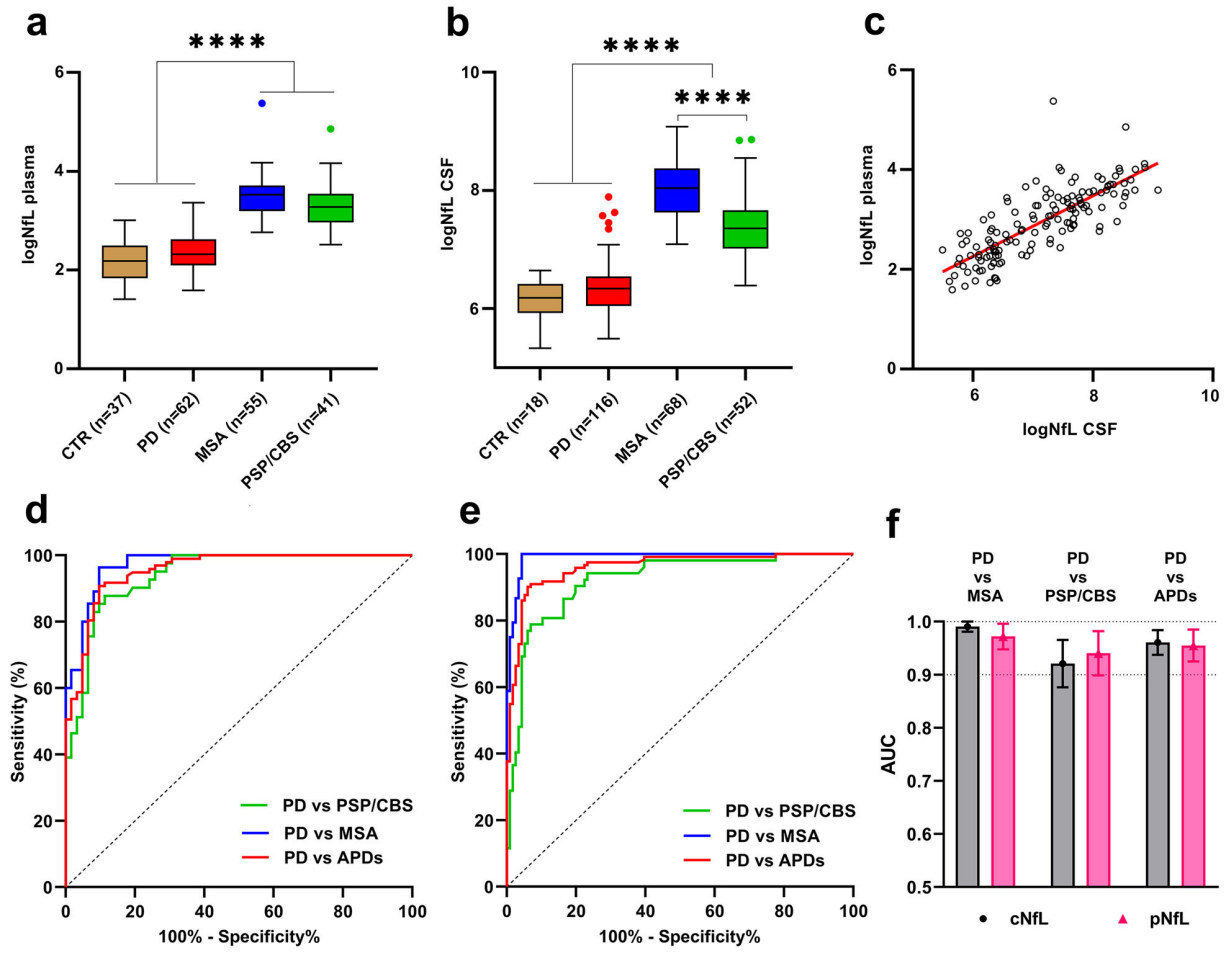
Plasma and CSF NfL values and ROC analysis to discriminate between PD and APDs. Boxplots illustrate natural-log transformed levels of **a** pNfL and **b** cNfL in the main diagnostic groups; ****p < 0.0001. Bounds of box plots show the range from the 25th to the 75th percentiles and the central line indicate the median value of the distribution; whiskers identify the Tukey’s range and symbols indicate the outliers according to the Tukey test. **c** Correlation between natural-log transformed pNfL and cNfL levels. ROC curves of **d** pNfL and **e** cNfL levels to distinguish PD from MSA, PSP/CBS, or the whole APDs group with **f** related actual AUC values (error bars indicate the 95% CI).

Patients with MSA and those with PSP/CBS showed higher levels of pNfL than controls and patients with PD (*p* < 0.0001 for both comparisons). Moreover, patients with MSA showed slightly higher pNfL values than those with PSP/CBS (*p* = 0.03) (Fig. 1a, Table 2).

**Table 2:** Plasma and CSF NfL values, and CSF Alzheimer’s disease biomarkers in the diagnostic groups.

	PD	PDD	DLB	MSA	PSP/CBS	iAF	iRBD	Controls
cNfL	566.5 (424.0–694.0)^a–d^	807.5 (643.8–1065.0)^b–e^	945.0 (734.3–1253.0) ^b–f^	3098.0 (2062.0–4314.0)^c,g–i^	1569.0 (1120.0–2128.0)^g–i^	548.0 (448.0–791.5)	675.0 (468.0–781.0)	514.2 (339.0–650.0)
pNfL	10.2 (8.1–13.8)^b–d,j,k^	15.9 (12.6–20.2)^b,c^	17.1 (14.7–34.2)^b,d,l^	34.0 (24.5–40.8)^g–i,l^	26.6 (19.4–40.8)^h,i^	15.8 (14.2–24.9)^f,i^	15.1 (7.8–16.2)	8.9 (6.3–12.2)
t-tau	172.0 (127.5–221.5)^b,f^	183.0 (110.3–287.3)^b^	236.0 (184.0–338.0)^m^	251.5 (177.0–320.8)^i,l,n^	195.0 (157.3–282.3)	185.0 (138.5–273.0)	205.0 (150.5–293.5)	182.0 (143.0–218.0)
p-tau	26.0 (21.0–33.0)	29.0 (21.0–43.8)	34.5 (27.0–48.0)	25.5 (21.0–36.0)	29.0 (22.3–37.8)	29.0 (22.0–34.5)	28.0 (21.5–43.5)	28.0 (22.3–38.0)
Aβ42	691.0 (527.5–924.0)^a,o^	526.0 (366.8–710.0)^f^	547.5 (355.5–748.5)^f,l^	583.0 (409.8–808.8)^e,p^	655.0 (476.3–877.0)	749.0 (506.5–1015.0)	766.0 (570.0–1241.0)^d^	692.0 (494.0–1068.0)
Aβ40	8424.0 (6134.0–10649.0)^a,b^	7305.0 (4781.0–11298.0)^f^	7733.0 (6566.0–9590.0)^p^	6625.0 (4947.0–9108.0)^e,p^	8304.0 (5761.0–10664.0)^f^	9552.0 (6688.0–11272.0)^d^	8413.0 (7544.0–13829.0)^q^	7846.0 (6040.0–10047.0)
Aβ42/40	0.86 (0.79–0.91)^a,q^	0.78 (0.59–0.88)^r^	0.79 (0.53–0.93)^l^	0.88 (0.80–0.93)	0.86 (0.77–0.94)	0.85 (0.73–0.93)	0.85 (0.77–0.94)	0.94 (0.72–1.26)

Data are expressed as median (interquartile range). CSF markers were analysed in 116 PD, 36 PDD, 64 DLB, 68 MSA, 52 PSP/CBS, 29 iAF, 19 iRBD and 35 controls. Plasma markers were analysed in 62 PD, 18 PDD, 33 DLB, 54 MSA, 40 PSP/CBS, 14 iAF, 7 iRBD and 37 controls. All biomarkers (ln-transformed) were analyzed with univariate general linear models adjusting for age: ^a^compared to PDD ≤ 0.05; ^b^compared to MSA ≤ 0.0001; ^c^compared to PSP/CBS ≤ 0.0001; ^d^compared to controls ≤ 0.05; ^e^compared to iAF ≤ 0.05; ^f^compared to iRBD ≤ 0.05; ^g^compared to iAF ≤ 0.0001; ^h^compared to iRBD ≤ 0.0001; ^i^ compared to controls ≤0.0001; ^j^ compared to DLB ≤ 0.05; ^k^compared to iAF ≤ 0.001; ^l^ compared to PSP/CBS ≤ 0.05; ^m^ compared to MSA ≤ 0.05; ^n^ compared to iAF ≤ 0.01; ^o^ compared to MSA ≤ 0.01; ^p^compared to iRBD ≤ 0.01; ^q^compared to controls ≤ 0.01; ^r^compared to PSP/CBS ≤ 0.01.

*cNfL* CSF neurofilament light chain; *pNfL* plasma NfL, *t-tau* total-tau, *p-tau* phospho-tau, *Aβ* amyloid-beta.

The concentration of cNfL showed a similar trend to that of pNfL, with the MSA and PSP/CBS groups having significantly higher levels than those of the patients with PD (*p* ≤ 0.0001 for both comparisons). Additionally, cNfL levels were significantly higher in the MSA group than in the PSP/CBS group (*p* < 0.0001) (Fig. 1b, Table 2). There were no significant differences between MSA patients with predominant cerebellar ataxia (MSA-C), predominant parkinsonism (MSA-P), or presenting with isolated AF (Supplementary Tables 1 and 2). Moreover, a comparison between MSA patients with the biofluid examined within the first three years of the clinical course (*n* = 30) or afterwards (*n* = 50) did not show any statistically significant difference (Supplementary Table 3).

In the PD and APD (MSA+PSP/CBD) patients with both CSF and blood samples tested, pNfL correlated strongly with cNfL (*r* = 0.82, *p* < 0.0001) (Fig. 1c).

ROC curves analysis demonstrated high accuracy for both cNfL and pNfL in the discrimination of PD from MSA (CSF: AUC 0.991, 95.7% sensitivity, 100% specificity, cut-off 1196 pg/mL; plasma: AUC 0.972, 90.3 sensitivity, 96.4% specificity, cut-off 17.2 pg/mL) and PSP/CBS (CSF: AUC 0.940, 97.4% sensitivity, 80.8% specificity, cut-off 1057 pg/mL; plasma: AUC 0.936, 88.7% sensitivity, 87.8% specificity, cut-off 16.6 pg/mL) (Fig. 1d–f, Table 3).

**Table 3:** Sensitivity and specificity of the NfL and α-syn RT-QuIC assays in patients with PD and APDs.

Diagnostic Group	Biomarker	NfL cut-off (pg/ml)	+/− ^1^	Sens. (%) [95% CI]	Spec. (%) [95% CI]
PD	α-syn-s	–	106/10	91.4 [84.7–95.8]	–
MSA	α-syn-s	–	3/65	–	95.6 [87.6–99.1]
PSP/CBS	α-syn-s	–	0/52	–	100 [93.1–100]
APDs	α-syn-s	–	3/117	–	97.5 [92.9–99.5]
Controls	α-syn-s	–	1/34	–	97.1 [85.1–99.9]
PD vs MSA	cNfl	1196.0	111/5 vs 0/68	95.7 [90.3–98.2]	100 [94.7–100]
	pNfL	17.2	56/6 vs 2/53	90.3 [80.5–95.5]	96.4 [87.7–99.4]
	cNfL + α-syn-s^a^	1196.0	–	99.1 [95.3–99.9]	95.6 [87.6–99.1]
PD vs PSP/CBS	cNfl	1057.0	108/8 vs 10/42	97.4 [92.6–99.5]	80.8 [68.1–89.2]
	pNfL	16.6	55/7 vs 5/36	88.7 [78.5–94.4]	87.8 [74.5–94.7]
	cNfL + α-syn-s^a,b^	595.0	–	97.4 [92.6–99.5]	100 [93.2.–100]
PD vs APDs	cNfL	1083.0	109/7 vs 11/109	93.9 [88.1–97.1]	90.8 [84.3–94.8]
	pNfL	17.2	56/6 vs 8/88	90.3 [80.5–95.5]	91.7 [84.2–96.3]
	cNfL + α-syn-s^a,c^	700.0	–	98.3 [93.9–99.8]	95.8 [90.5–98.6]

^1^ “+” for α-syn seeds indicates a positive seeding reaction in the RT-QuIC analysis, while it means “below the cut-off” for the NfL assay. Conversely, “−“ for α-syn seeds indicates a negative seeding reaction in the RT-QuIC analysis, and a “above the cut-off” value of NfL.

^a^*p* < 0.05 when compared to RT-QuIC alone.

^b^*p* ≤ 0.001 when compared to cNfL alone.

^c^*p* < 0.05 when compared to cNfL alone.

*Sens.* sensitivity, *Spec.* specificity.

### Value of CSF NfL in combination with α-syn RT-QuIC in the differential diagnosis of patients with parkinsonism

**Fig. 2:**
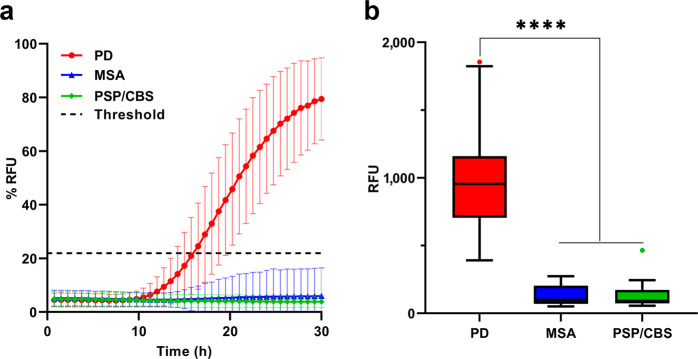
Comparison of RT-QuIC seeding activity in PD and APDs. **a** Kinetic curves of PD patients with positive seeding activity compared to those of (all) patients from PSP/CBS, and MSA groups. Each curve represents the average of the group, error bars indicate the SD. **b** The area under the curve was chosen as the most representative parameter describing the kinetics of α-syn aggregation in the RT-QuIC assay. Bounds of box plots show the area under the curve range from the 25th to the 75th percentiles and the central line indicate the median value of the distribution; whiskers identify the Tukey’s range and symbols indicate the outliers according to the Tukey test.

As we previously showed in a smaller cohort^12^, positive seeding activity was consistently detected only in patients with PD (106/116, 91.4%). In contrast, only three out of 68 (4.4%) cases in the MSA group yielded a positive RT-QuIC result, and no seeding activity was detected in the PSP/CBS group (Table 3, Fig. 2).

For the discrimination of PD from APDs, the sensitivity of the combined test was higher than that of the tests evaluated individually (91.4% RT-QuIC; 93.9% cNfL; 98.3% combined), whereas the specificity increased compared to that of cNfL assay alone and decreased only slightly compared to that of RT-QuIC test alone (97.5% RT-QuIC; 90.8% cNfL; 95.8% combined, *p* = 0.047 vs. RT-QuIC alone and *p* = 0.011 vs. NfL alone) (Table 3). Similarly, the combined tests showed highest diagnostic accuracy for the discrimination between PD and PSP/CBS (AUC 0.99; *p* = 0.007 vs. RT-QuIC; *p* = 0.0001 vs. cNfL) and between PD and APDs (AUC 0.97; *p* = 0.047 vs. RT-QuIC; *p* = 0.011 vs. cNfL), whereas for PD vs. MSA (AUC 0.97) the combination performed better than the RT-QuIC alone (*p* = 0.002), but not compared to the cNfL alone (*p* = 0.751) (Table 3). Results of the combined test analysis are summarized in Supplementary Table 4. Details about patients with discrepant results despite the combined analysis are provided in Supplementary Table 5.

### NfL biomarker and α-syn RT-QuIC in iAF and iRBD syndromes

Patients with iRBD or iAF showed cNfL levels comparable to those of PD patients (Table 2). However, patients with iAF had significantly higher pNfL levels compared to those of patients with PD (*p* = 0.001), whereas, pNfL levels did not differ significantly between the iRBD and PD groups.

As we showed in a previous study^12^, most patients with iRBD and iAF in our cohort had positive results by α-syn RT-QuIC, with the exception of three patients with iAF (26/29, 89.7%) and one with iRBD (18/19, 94.7%).

### NfL, α-syn seeding activity, and AD core markers in patients with parkinsonism and cognitive decline

Patients with PDD showed significantly lower pNfL concentrations than those of patients with MSA or PSP/CBS (*p* < 0.0001 for both comparisons), and no differences with PD patients. In contrast, PDD patients showed higher cNfL levels than those of PD group (*p* = 0.039), and significantly lower values than those of MSA or PSP/CBS groups (*p* < 0.0001 for both comparisons) (Table 2).

pNfL levels in the DLB group were significantly lower than those in the MSA group (*p* = 0.001), but higher than those in the PD and control groups (*p* = 0.05 and *p* = 0.012, respectively). Similarly, cNfL concentrations in DLB group were lower than those in the MSA and PSP/CBS groups (*p* < 0.0001 for both comparisons), while statistically significance was not reached in comparison with the PD group. Finally, there were no differences between PDD and DLB groups in either pNfL or cNfL levels.

As shown previously^12^, α-syn RT-QuIC assay detected a positive seeding reaction in most patients with PDD (35/36, 97.2%) and DLB (63/64, 98.4%). The diagnostic values for pNfL, cNfL, and α-syn RT-QuIC in the differential diagnosis between PDD or the combined PD/PDD groups and patients with APDs are shown in Supplementary Table 6.

Analysis of CSF AD core biomarkers revealed a higher proportion of A+ [amyloid-beta (Aß) positive] and T+ (phospho-tau positive) cases in the DLB and PDD groups than in the PD, MSA, and, to a lesser extent, PSP/CBS groups (Table 4), which correlated with lower MMSE scores in the former groups (Table 1). Overall, the frequency of N+ (neurodegeneration) cases was low in all groups. Notably, the only PD patient with an A+T+N+ profile showed cognitive decline two years after CSF collection and eventually received a PDD diagnosis. In the other 6 PD patients who developed dementia 3 to 9 years after LP, the A/T/N status was unremarkable.

**Table 4:** Distribution of AD co-pathology according to the A/T/N CSF profile in the main diagnostic groups.

A/T/N	PD	PDD	DLB	PSP/CBS	MSA	*p* value^b^
status^a^	(*n* = 116)	(*n* = 36)	(*n* = 64)	(*n* = 52)	(*n* = 68)	
A+T+N+	1(0.9)	1(2.8)	4(6.3)	1(1.9)	1(1.5)	0.232
A+T+N−	0(0.0)	2(5.5)	5(7.8)	1(1.9)	0(0.0)	0.007
A+T−N-	6(5.2)	7(19.4)	18(28.1)	4(7.7)	6(8.8)	<0.0001
A−T+/−N+/−	109(93.9)	26(72.2)	37(57.8)	46(88.5)	61(89.7)	<0.0001

Data are expressed as *n* (%).

^a^A/T/N classification according to the following criteria: A+ Aβ42/40 ratio <0.65, T+ p-tau >58 pg/ml, N+ t-tau >450 pg/ml. The A+/T−/N+ profile was not observed.

^b^A+T+N−: DLB vs PD 0.005, DLB vs MSA 0.025. A+T−N−: DLB vs PD < 0.0001, DLB vs PSP/CBS 0.008, DLB vs MSA 0.006, PDD vs PD 0.014. Amyloid status (A+ vs A−): DLB vs PD < 0.0001, DLB vs PSP/CBS 0.0004, DLB vs MSA < 0.0001, PDD vs PD 0.001, PDD vs MSA 0.028.

### Association of plasma and CSF NfL levels with disease severity and survival across diagnostic groups

Given that PDD represents an evolutionary stage of PD related to the spread of LB pathology and/or the association of AD or other co-pathologies, we considered PD and PDD as a single entity when evaluating the NfL association with disease severity. In the PD/PDD cohort, both pNfL and cNfL levels were significantly associated with motor impairment, measured by clinical scales, the MMSE score, or the presence of orthostatic hypotension, while cNfL levels but not pNfL levels positively correlated with disease duration. However, only the association between cNfL levels and motor scores remained significant after adjusting for age (Supplementary Tables 7 and 8).

Additionally, levels of pNfL, but not of cNfL in MSA patients, were significantly associated with orthostatic hypotension in both uni- and multivariate models.

In the PD/PDD cohort, both pNfL and cNfL were significantly higher in CSF A+ patients, and a trend towards a negative correlation between pNfL levels and the amyloid ratio (data not shown) was seen, but this correlation was not statistically significant after adjusting for age.

Lastly, univariate Cox regression analyses showed an association between cNfL level and survival in the PD/PDD (HR = 12.6, *p* = 0.01) and DLB (HR = 4.05, *p* = 0.004) groups. However, the effect was no longer statistically significant after correcting for age and disease duration. There were no significant associations between cNfL and survival in the remaining cohorts and between pNfL level and survival in any patient group (data not shown).

## Discussion

Here we reported the results of a comprehensive evaluation of the diagnostic and prognostic value of NfL and α-syn seeding activity, including the comparison between cNfL and pNfL performance, and the analysis of the added value of a combined CSF analysis of NfL and α-syn seeding activity, in a large cohort of patients with parkinsonism and related prodromal syndromes.

In line with the results of previous studies^6,7,9,10^, we found that cNfL levels can distinguish between patients with PD and those with APDs with high diagnostic accuracy (sensitivity 93.9%; specificity 90.8%, AUC 0.97). Additionally, given the strong correlation between plasma and CSF NfL levels, the diagnostic accuracy of pNfL was as high as that of cNfL (sensitivity 90.3%; specificity 91.7%, AUC 0.96). Notably, our CSF results showed AUC values in the upper range of those published to date (range 0.90–0.97)^6,9,10,17^, while those for plasma yielded the highest AUC ever published (range 0.80–0.91)^6,8,10^. In this regard, the difference in NfL levels between PD and MSA patients was particularly significant in our cohort, which is one of the largest to be studied till date. The comparison between MSA patients with the biofluid examined within the first three years of the clinical course or afterwards excluded the effect of the timing of biofluid sampling. Moreover, one patient in the iAF group and one in the iRBD group progressed to MSA during the follow-up, and in these two patients, the levels of both cNfL and pNfL were already significantly increased and above the cut-off level in the prodromal stage. Taken together, these findings suggest that NfL levels increase significantly in MSA patients during the early clinical disease stages.

By expanding the patient cohort, we confirmed the previously reported difference in α-syn reaction kinetics between APDs and LBD samples^12^. Indeed, among patients with APDs, only 4.4% of the MSA cases showed α-syn seeding activity, and only 8.6% of patients with PD showed a negative RT-QuIC assay result. This resulted in a 91.4% sensitivity and 97.5% specificity for α-syn seeding activity in distinguishing PD from APDs. Combining the two assays and requiring for PD diagnosis NfL levels to be lower than the chosen cut-off and α-syn seeds to be ‘positive’ provided a test with 98.3% sensitivity and 95.8% specificity in separating PD from APDs. Similarly, a significant improvement of accuracy was detected by combining the two tests in the discrimination between PD and PSP/CBS (sens. 97.4%, spec. 100%). In contrast, in the comparison between PD and MSA, the combined tests improved the accuracy only against the RT-QuIC alone, not against cNfL alone, reflecting the highly significant difference in cNfL levels between the two diagnostic groups. Overall, combining these markers for the diagnostic evaluation of patients with parkinsonism provides some additional accuracy and may have confirmatory value in front of a borderline or inconclusive result in one test.

The results of the NfL level evaluation in patients with iRBD and iAF deserve a further comment. In both groups, patients with a positive CSF α-syn RT-QuIC assay result showed low cNfL levels, comparable with those of patients with PD. In contrast, patients with iAF showed higher pNfL values than those of patients with iRBD or PD, suggesting a contribution of peripheral autonomic nerve degeneration to pNfL values. Consequently, cNfL levels appear to be more accurate than pNfL levels in the prediction of phenoconversion to PD, DLB or MSA in patients showing only AF at onset.

Higher cNfL level in the PD/PDD group was positively associated with motor scores after adjusting for the effect of age. In the MSA cohort, pNfL levels slightly positively correlated with the presence of orthostatic hypotension, whereas no significant associations were found in the PSP/CBS and DLB groups. Thus, our results confirm that both cNfL and pNfL concentrations might represent markers of disease severity in PD/PDD, reflecting the intensity of the neurodegenerative process, which could be of importance in future clinical trials^18–21^. The lack of a significant association between NfL values and motor impairment in the MSA and PSP/CBS groups likely depends on the more severe and rapidly progressing neurodegeneration seen in these diseases compared to that in PD^22^. However, the fact we used the UPDRS-III and Hoehn and Yahr motor scales, which best reflect the motor impairment of a classic parkinonian syndrome and are not ideal for a multisystem disorder such as MSA, could also provide an explanation for this finding. Finally, the lower number of patients analysed compared to that in the PD group should also be considered.

As expected, we found a significantly higher percentage of patients with an abnormal CSF AD-related A/T/N profile in DLB or PDD than in the other diagnostic groups. Moreover, there were no significant differences in the prevalence of CSF AD core markers between groups in the absence of cognitive decline. Using an automated platform and a validated reference standard for Aβ42 measurement in a large cohort, we documented that approximately 1 out of 10 of the unselected patients with neurodegenerative parkinsonism has an abnormal Aβ42/40 ratio, which is considered the most accurate proxy CSF biomarker for AD-related brain amyloid status^23^.

It is foreseeable that all the markers tested in the present study will have critical roles in clinical trials of novel drug candidates against LB α-syn pathology in PD^24^. The RT-QuIC assay can be used to select patients with positive α-syn seeding activity and for effective drug monitoring. Moreover, NfL levels and AD biomarkers could be used to rule out significant neurodegeneration and comorbid AD pathology. In this respect, the added value of CSF sample analysis in comparison to blood and other tissue samples such as the skin, which also provide accurate assessment of NfL (blood) and pathological α-syn (skin)^25,26^, must be emphasised. Indeed, CSF is to date the only single source allowing the combined assessment of NfL levels, α-syn seeding activity, and AD core markers.

The present study’s main limitation is that we used clinical criteria and not neuropathological data for the study participants’ diagnoses. However, medical doctors specialized in movement disorders established the clinical diagnoses in patients followed over time with reassessments at each follow-up visit. Moreover, we included only patients fulfilling the criteria for probable or clinically established disease. These selection criteria probably minimised the discrepancy between clinical and post-mortem diagnosis. Another limitation relies on the cross-sectional design which makes it difficult to assess the association between a marker (i.e., NfL) and clinical end points in individual patients. Additional prospective studies in which the biomarkers are analysed at disease onset and the diagnosis is established after a long clinical follow-up or post-mortem are needed to validate the value of NfL level and α-syn seeding activity as early diagnostic markers. Furthermore, the value of NfL as a marker of disease progression should be evaluated serially through multiple assessments in individual patients over the disease course.

In conclusion, we demonstrated the value of individual and combined assessment of levels of pNfL and cNfL and CSF α-syn seeds by RT-QuIC as diagnostic markers in a single large cohort of patients with neurodegenerative parkinsonism. Moreover, we contributed cNfL and pNfL values in patients with iAF and iRBD. By showing that each of the three biomarkers accurately distinguishes between PD and APDs, with the combination of cNfL and α-syn seeds providing the highest diagnostic value, our results confirm and expand on previous studies on the clinical value of these biomarkers for patients with parkinsonism and related syndromes.

## Methods

### Ethical approval

The study was approved by the ethics committee “Area Vasta Emilia Centro” (approval numbers AVEC: 09070, 17093, 18025, and 18027). Written informed consent was given by study participants or by the next of kin for patients unable to communicate due to mental impairment.

### Study populations and inclusion criteria

The cohort included 355 patients affected by parkinsonism of probable neurodegenerative etiology diagnosed by a movement disorder specialist at the Institute of Neurological Sciences of Bologna (ISNB) between 2007 and 2019. We selected cases with a probable clinical diagnosis at last follow-up and available plasma and/or CSF samples. Of the 355 subjects, 336 had CSF, and 213 blood. 61 patients belonged to the *BOPROPARK* cohort^27^.

We also analyzed blood and CSF samples from 49 patients affected by syndromes that may precede the onset of parkinsonism, such as iAF (*n* = 30, all from the *IAF-BO* cohort^28^), and iRBD (*n* = 19), and from 72 non-neurodegenerative controls. The latter included plasma samples from 37 healthy subjects and CSF samples from 35 subjects lacking any clinical or neuroradiological evidence of disease affecting the nervous system (i.e., patients with chronic migraine, subjective cognitive decline, and mild psychiatric symptoms). Approximately half of the patients (*n* = 201) were included in a previous study reporting results of α-syn RT-QuIC analysis^12^.

### Clinical assessment

For motor assessment, we used the Unified Parkinson’s Disease Rating Scale (UPDRS-III), and the Hoehn and Yahr staging. Cognition was assessed using the Mini-Mental State Examination (MMSE). Diagnostic investigations included, when available, brain magnetic resonance imaging (MRI, *n* = 239), cerebral 129I-ioflupane SPECT (DaTSCAN) (*n* = 181), cardiac 123I-metaiodobenzylguanidin (MIBG)-SPECT (*n* = 95), and all-night polysomnography (PSG, *n* = 243). All patients with suspected AF (*n* = 90) were assessed by a battery of cardiovascular reflex tests, including head-up tilt test (10 min at 65°), Valsalva maneuver (40 mm Hg for 15 s), deep breathing (6 breaths/min), and sustained handgrip (one-third of maximal effort for 5 min). After CSF collection, most patients were longitudinally followed-up for more than one year (241 cases, 59.7% of total cases) and 135 of them for more than three years (33.4% of total cases). The clinical diagnosis formulated at baseline were reevaluated at each follow-up visit. Only patients with a “probable” or “clinically established” (for PD only) diagnosis at last follow-up of iRBD^29^, iAF^30,31^, PD^32^, MSA^1^, PSP/CBS^33,34^, PDD^35^, and DLB/prodromal DLB^36,37^, according to internationally established criteria were included in the study cohort. The term isolated iRBD indicated the absence of any associated neurological sign or secondary causes of the sleep disturbance^38^.

### Blood and CSF biomarker analyses

We performed all blood analyses on a single molecule array (Simoa) SR-X analyzer platform (Quanterix, Billerica, MA, USA). Plasma NfL was measured with Simoa NF-light advantage kits. The mean intra- and inter-assay coefficients of variation (CVs) for pNfL were 4 and 11%.

CSF total-tau (t-tau), p-tau, Aβ42, and Aβ40 were measured by automated chemiluminescent enzyme-immunoassay (CLEIA) Lumipulse assays (Fujirebio Europe NV, Gent, Belgium). The inter-assays CVs were ˂8% for all biomarkers. The Aβ42/40 ratio was calculated as published^39^.

NfL in CSF samples was quantified by a validated commercial enzyme-linked immunosorbent assay (NfL ELISA kit, IBL, Hamburg, Germany)^40^. The mean intra- and inter-assays CVs were 2 and 10%.

We performed the α-syn RT-QuIC assay as described^12^, with minor modification. To limit the possible batch-to-batch variations of α-syn activity and the intrinsic plate-to-plate experimental variability, we run the same positive control throughout all experiments, and normalized the relative fluorescent units (RFU) for every time point for the maximum intensity reached by the positive control. Samples were run in quadruplicates and deemed positive when at least 2 out of 4 replicates reached the threshold. The latter was calculated as the average normalized fluorescence value of negative control replicates during the first 10 h, plus 30 standard deviations. The cut-off was set at 30 h. When only one replicate crossed the threshold, the analysis was considered “unclear” and repeated up to three times. All biomarker analyses were performed by personnel blinded to the clinical diagnostic group. RT-QuIC results in 201 (48%) subjects were previously reported^12^.

### Statistical analyses

NfL concentrations in both CSF and plasma were natural-log transformed to fulfill the normal distribution and all statistical analyses were performed on transformed data. Student *t*-test, Mann–Whitney *U* test, one-way analysis of variance (followed by Tukey’s post hoc test) and Kruskal–Wallis test (followed by Dunn’s post hoc analysis) were used to compare the continuous variables between the groups. We adopted the Chi-Square test for categorical variables. Natural logarithm of cNfl, pNfL, and Alzheimer disease (AD) CSF biomarkers (dependent variable) were compared between diagnostic groups (independent variable) with multivariate general linear models adjusting for age.

Receiver Operating Characteristic (ROC) analyses were performed, and the area under the ROC curve (AUC), the sensitivity and specificity with relative 95% confidence intervals (95% CI) were calculated to evaluate the diagnostic accuracy (PD vs MSA, PD vs PSP/CBS, PD vs APDs) of cNfL, pNfL, and α-syn RT-QuIC in CSF. The optimal cut-off value for NfL biomarker in both CSF and plasma was chosen using the Youden’s Index. To investigate the performance of the cNfL and α-syn RT-QuIC assays in combination, the two tests were also analyzed in parallel. Before combining them, we chose a new cut-off for cNfL that maximizes the specificity. Then, a diagnosis of PD was given when at least one of the two tests was positive (i.e., cNfL lower than the cut-off value and/or positive α-syn RT-QuIC seeding activity), while that of APDs was given to those yielding a negative result (for PD) in both tests. De Long test was used to compare the AUC between the tests performed individually and the combined test. Differences were considered statistically significant at *p*-value < 0.05.

Spearman’s correlation (rho), univariate and multivariate linear regression models adjusted for age were used to evaluate the association of clinical variables with pNfL and cNFL values, stratified for the diagnostic groups. The results are presented as Beta coefficients (β) and 95% CI^21^.

The cumulative time-dependent probability of death from LP was calculated by the Kaplan–Meier estimate. We performed univariate and multivariate Cox regression analyses to test the association between survival and continuous values or tertiles of each biomarker and known prognostic factors. The results are presented as Hazard Ratios (HR) and 95% CI, stratified for the diagnostic groups.

Further details on methods are provided in Supplementary Methods.

### Reporting summary

Further information on research design is available in the Nature Research Reporting Summary linked to this article.

## Supplementary information

Supplementary Information

Reporting summary

## Data Availability

The data that support the findings of this study are available from the corresponding author upon reasonable request.
