# Supplementary material for: Neurofilament light chain and α-synuclein RT-QuIC as differential diagnostic biomarkers in parkinsonisms and related syndromes
Source: NPJ Parkinsons Dis. 2021 Oct 11;7:93. doi: 10.1038/s41531-021-00232-4 (PMC8505434; doi:10.1038/s41531-021-00232-4)
Supplement: Supplementary file 1 — Supplementary Information [file 41531_2021_232_MOESM1_ESM.pdf]

Supplementary Materials for the manuscript:

Neurofilament light chain and α-synuclein RT-QuIC as differential diagnostic biomarkers in parkinsonisms and related syndromes

Corinne Quadalti, Giovanna Calandra-Buonaura, Simone Baiardi, Andrea Mastrangelo, Marcello Rossi, Corrado Zenesini, Giulia Giannini, Niccolò Candelise, Luisa Sambati, Barbara Polischi, Giuseppe Plazzi, Sabina Capellari, Pietro Cortelli, Piero Parchi.

Supplementary Methods

Sample collection

EDTA plasma samples were collected, aliquoted, and stored at −80°C according to standard procedures. CSF samples were obtained by lumbar puncture (LP) following a standard procedure, centrifuged in case of blood contamination (<2% of cases), divided into aliquots, and stored in polypropylene tubes at −80 °C until analysis.

A/T/N classification

We classified all cases with available CSF according to the A/T/N scheme, [1] as follow: amyloid positive (A+), if Aβ42/40 ratio <0.68; tau positive (T+), if p-tau >58 pg/ml; neurodegeneration positive (N+), if t-tau >450 pg/ml.

Statistical analysis and patient group comparisons.

We used IBM SPSS Statistics version 24 (IBM, Armonk, NY, USA) and GraphPad Prism 8 (GraphPad Software, La Jolla, CA, USA) software. Depending on their distribution, data were presented as mean ± standard deviation (SD) or median and interquartile range (IQR).

In both linear regression models and Cox regression analyses amyloid ratio and orthostatic hypotension were analysed as categorical parameters (presence or absence). Positive amyloid status was defined by a ratio <0.65, whereas the presence of orthostatic hypotension was defined by a drop in systolic blood pressure of ≥20 mmHg or diastolic blood pressure of ≥10 mmHg with assumption of an upright posture.[2]

In this study PD and PDD groups have been considered two different cohorts, except when evaluating the association with disease severity of cNfL and pNfL. We kept the two groups separate when assessing the diagnostic value of NfL because the presence or absence of cognitive decline makes a difference in the early diagnostic evaluation of these patients in the clinical setting. Moreover, PDD is by definition an evolutionary stage of PD with cognitive decline occurring at least one year after the onset of parkinsonism. In contrast, given that PDD represents an evolutionary stage of PD related to the spread of LB pathology and/or the association with AD pathology or other co-pathologies, we considered PD and PDD as a single entity when evaluating NfL association with disease severity.

Supplementary References

[1] Jack, CR Jr., et al. An unbiased descriptive classification scheme for Alzheimer disease biomarkers. *Neurology*, 87(5), 539-47, (2016).

[2] Freeman, R., et al. Consensus statement on the definition of orthostatic hypotension, neurally mediated syncope and the postural tachycardia syndrome. *Clin. Auton. Res.* 21(2), 69-72, (2011).

Supplementary Table 1. Comparison of NfL levels and clinical/demographic features between MSA with predominant cerebellar ataxia (MSA-C) and MSA with predominant parkinsonism (MSA-P).

	**MSA-C**	**MSA-P**	**p value**
**n**[1]	44	34	
**Age, yrs**	60.5±7.2	61.9±9.1	0.449
**Female, n (%)**	20 (45.5)	22 (64.7)	0.091
**Time between onset to sample collection, mo.**	43.0 (32,0-61.3)	50.5 (28.0-68.3)	0.539
**CSF NfL (pg/ml)**	3151 (2153-4487)	3119 (1932-4281)	0.567
**Plasma NfL (pg/ml)**	29.1 (22.3-38.6)	35.8 (26.8-47.6)	0.084
**Orthostatic hypotension (%)**	21 (47.7)	26 (68.4)	0.059
**Aβ42/40 <0.65 (%)**	5 (11.9)	2 (6.9)	0.693

Age is expressed as mean±SD, while the other continuous variable as median (interquartile range). CSF NfL was analysed in 38 MSA-C and 29 MSA-P, while plasma NfL in 30 MSA-C and 23 MSA P. [1]Two patients were excluded from the analysis because of the equal severity of parkinsonian and cerebellar features.

Supplementary Table 2. Comparison of NfL levels and clinical/demographic features between MSA presenting with isolated autonomic failure and MSA with other symptoms/signs at onset.

**Clinical presentation**	**isolate autonomic failure**	**other symptoms/signs**	**p value**
**n**	18	62	
**Age, yrs**	61.6±6.9	61.0±8.4	0.784
**Female, n (%)**	9 (50,0)	34 (54.8)	0.792
**Time between onset to sample collection, mo.**	45.5 (34.5-68.0)	43.0 (25.8-67.5)	0.619
**CSF NfL (pg/ml)**	3249 (2408-4604)	3009 (1995-4266)	0.618
**Plasma NfL (pg/ml)**	27.6 (23.9-24.5)	35.1 (24.5-41.5)	0.251

**MSA-C/MSA-P (%)**	61.1/38.9	55.0/45.0	0.788
**Aβ42/40 <0.65 (%)**	13.3	9.4	0.645

Age is expressed as mean±SD, while the other continuous variable as median (interquartile range). CSF NfL was analyzed in 15 MSA-isolate AF and 53 MSA-other symptoms/signs at onset, while plasma NfL in 12 MSA-isolate AF and 43 MSA-other symptoms/signs at onset.

Supplementary Table 3. Comparison of NfL levels and clinical/demographic features in MSA between early (<3 years from onset) and late disease stages.

	**MSA early (≤3 yrs)**	**MSA late (>3 yrs)**	**p value**
**n**	30	50	
**Age, yrs**	59.3±7.8	62.3±8.0	0.108
**Female, n (%)**	13 (43.3)	30 (60.0)	0.149
**Time between onset to sample collection, mo.**	25.0 (20.3-32.3)	59.0 (45.3-72.8)	<0.0001
**CSF NfL (pg/ml)**	3098 (2018-4520)	3075 (2118-4249)	0.768
**Plasma NfL (pg/ml)**	31.1 (21.3-37.9)	34.6 (25.2-42.3)	0.393
**Orthostatic hypotension (%)**	22 (73.3)	38 (79.2)	0.552
**Aβ42/40 <0.65 (%)**	2 (7.7)	5 (11.9)	0.699

Age is expressed as mean±SD, while the other continuous variable as median (interquartile range). CSF NfL was analysed in 26 MSA early and 42 MSA late, while plasma NfL in 17 MSA early and 38 MSA late.

Supplementary Table 4. Summary of the results of the cNfL and α-syn RT-QuIC combined analysis between diagnostic groups.

**PD vs MSA**		**PD Diagnosis**	
		**Y**	**N**
**cNfL + α-syn-s**	**+**	115	3
	**-**	1	65
	**TOT**	116	68
**ROC AUC [95% CI]**		0,974 [0,948–0,999]	

**PD vs PSP/CBS**		**PD Diagnosis**	
		**Y**	**N**
**cNfL + α-syn-s**	**+**	113	0
	**-**	3	52
	**TOT**	116	52
**ROC AUC [95% CI]**		0.987 [0.973-1.000]	

**PD vs APDs**		**PD Diagnosis**	
		**Y**	**N**
**cNfL + α-syn-s**	**+**	114	5
	**-**	2	115
	**TOT**	116	120
**ROC AUC [95% CI]**		0.971 [0.949-0.992]	

List of abbreviations: PD Parkinson's disease; MSA multiple system atrophy; PSP/CBS progressive supranuclear palsy/corticobasal syndrome; APDs atypical parkinsonisms (i.e. MSA + PSP/CBS).

cNfL: CSF Neurofilament light chain; α-syn-s α-synuclein seeding activity.

Y patients with positive PD diagnosis; N patients with non-PD diagnosis (i.e. MSA, PSP/CBS, APDs).

"+" positive α-syn-s OR cNfL value below the selected cut-off for the combination; "-" negative α-syn-s AND cNfL value above the selected cut-off for the combination (see Table 3 of the manuscript for cut-offs values).

ROC AUC [95% CI] indicates the area under the ROC curve of the combined tests, with 95% confidence intervals.

Supplementary Table 5. Details regarding patients with discrepant results in PD vs APDs discrimination using both cNfL and α-syn RT-QuIC assays.

Diagnostic group	Final diagnosis	Age at onset	Time onset-LP	CSF NfL (pg/ml)	α-syn RT-QuIC result	Clinical features	Relevant diagnostic tests
**PD**	PD probable	69	38	691	Negative	Unilateral rest tremor accompanied by rigidity and bradykinesia. After 7 years from onset, falls and dysphagia. Unclear response to levodopa. No red flags.	MRI: mild vascular changes.
**PD**	PD probable	45	25	804	Negative	Rest tremor at onset. Subjective response to Levodopa. No red flags.	DatScan: reduced BG uptake.
**PD**	Clinically established PD	64	33	2674	Negative	Hyposmia. Unilateral bradykinesia and rigidity. Beneficial response to levodopa. Urinary urgency. No red flags.	MRI: moderate vascular changes.
**MSA**	MSA-P probable	57	71	3733	Positive	Parkinsonism poorly responsive to levodopa and early falls (<1 year). Then cerebellar and pyramidal signs. Urinary	MRI: cerebellar atrophy

						incontinence, orthostatic hypotension. RBD. Tracheostomy because of respiratory arrest during sleep.	
**MSA**	MSA-C probable	66	74	2712	Positive	Onset with cerebellar sign, then orthostatic hypotension, parkinsonism and pyramidal signs. RBD. Stridor. Urinary incontinence.	DatScan: reduced BG uptake; MIBG: abnormal adrenergic innervation
**MSA**	MSA-C probable	67	59	4608	Positive	Isolated autonomic failure (orthostatic hypotension, urinary incontinence) at onset, then parkinsonism, cerebellar and pyramidal signs. RBD. Stridor.	MRI: cerebellar atrophy; DatScan: reduced BG uptake; MIBG: normal adrenergic innervation
**PSP/CBS**	PSP-RS/P probable	59	67	596	Negative	Axial-predominant, akinetic-rigid parkinsonism, early falls. Then vertical gaze palsy, gait freezing, pyramidal signs, myoclonic jerks, and dysphagia.	MRI: vascular changes, fronto-temporal atrophy; DatScan: reduced BG uptake
**PSP/CBS**	PSP-RS/P probable	70	75	600	Negative	Axial-predominant, akinetic-rigid parkinsonism poorly responsive to levodopa,	MRI: mild vascular changes; DatScan:

						falls (<1 years), gait freezing, vertical gaze palsy, hypophonia, dysarthria, dysphagia, ideomotor apraxia, cervical dystonia.	reduced BG uptake

Discrepant results are highlighted in bold. Age is expressed in years, while the time between onset to lumbar puncture in months. List of abbreviations: LP, lumbar puncture; BG, basal ganglia; RBD, REM-sleep behavior disorder; MRI, magnetic resonance imaging; MIBG, metaiodobenzylguanidine.

Supplementary Table 6. Sensitivity and specificity of the NfL and α-syn RT-QuIC assay comparisons between PDD alone, and PD/PDD, versus atypical parkinsonisms.

Diagnostic Group	Biomarker	NfL cut-off (pg/ml)	+/- [1]	Sens. (%) [95% CI]	Spec. (%) [95% CI]
**PD/PDD vs MSA**	cNfl	1417	145/7 vs 1/67	95.4 [90.8-97.8]	98.5 [92.1-99.9]
	pNfL	17.43	71/13 vs 2/53	96.4 [87.7-99.4]	84.5 [75.3-90.7]
	α-syn-s	NA	142/10 vs 3/65	93.4 [88.2-96.8]	95.6 [87.6-99.1]
**PD/PDD vs PSP/CBS**	cNfl	1057	135/17 vs 10/42	80.8 [68.1-89.2]	88.8 [82.8-92.9]
	pNfL	17.56	72/12 vs 6/35	85.4 [71.6-93.1]	85.7 [76.7-91.6]
	α-syn-s	NA	142/10 vs 0/52	93.4 [88.2-96.8]	100 [93.2-100.0]
**PD/PDD vs APDs**	cNfL	1124	138/14 vs 13/107	90.8 [85.1-94.4]	89.2 [82.3-93.6]
	pNfL	17.56	72/12 vs 9/87	85.7 [76.7-91.6]	90.6 [83.1-95.0]
	α-syn-s	NA	142/10 vs 3/117	93.4 [88.2-96.8]	97.5 [92.9-99.5]
**PDD vs MSA**	cNfl	1417	34/2 vs 1/67	94.4 [81.9-99.0]	98.5 [92.1-99.9]
	pNfL	17.58	16/6 vs 3/52	94.6 [85.2-98.5]	72.7 [51.9-86.9]
	α-syn-s	NA	36/0 vs 3/65	100 [90.3-100.0]	95.6 [87.6-99.1]
**PDD vs PSP/CBS**	cNfl	1297	33/3 vs 16/36	69.2 [55.7-80.1]	91.7 [78.2-97.1]

	pNfL	17.56	16/6 vs 6/35	85.4 [71.6-93.1]	72.7 [51.9-86.9]
	α-syn-s	NA	36/0 vs 0/52	100 [90.3-100.0]	100 [93.2-100.0]
**PDD vs APDs**	cNfL	1364	34/2 vs 20/100	94.4 [81.9-99.0]	83.3 [75.7-88.9]
	pNfL	17.56	16/6 vs 9/87	90.6 [83.1-95.0]	72.7 [51.9-86.9]
	α-syn-s	NA	36/0 vs 3/117	100 [90.3-100.0]	97.5 [92.9-99.5]

List of abbreviations: PD, Parkinson's disease; MSA, multiple system atrophy; PSP/CBS, progressive supranuclear palsy/corticobasal syndrome; APDs, atypical parkinsonisms (MSA + PSP/CBS); Sens., sensitivity; Spec., specificity.

[1] "+" for α-syn seeds indicates a positive seeding reaction in the RT-QuIC analysis, while it means "below the cut-off" for the NfL assay. Conversely, "-" for α-syn seeds indicates a negative seeding reaction in the RT-QuIC analysis, and a "above the cut-off" value of NfL.

Supplementary Table 7. Associations between NfL values and clinical parameters (univariate model).

Diagnostic group	NfL source	Coefficient	Time onset to LP	MMSE	Hoehn & Yahr	UPDRS-III	Orthostatic hypotension	Amyloid ratio[1]
**PD/PDD**	CSF	B	0.002	-0.037	0.243	0.014	0.217	0.365
		95% CI	0.001-0.003	-0.059-0.015	0.130-0.356	0.006-0.022	0.021-0.413	0.141-0.590
		SE	0.001	0.011	0.057	0.004	0.099	0.114
		Beta	0.219	-0.281	0.378	0.353	0.192;	0.254
		P value	**0.007**	**0.001**	**<0.0001**	**0.001**	**0.030**	**0.002**
	Plasma	B	0.001	-0.400	0.200	0.009	0.362	0.418
		95% CI	-0.001-0.003	-0.064--	0.082-0.319	0.001-0.017	0.113-0.610	0.155-0.681
		SE	0.001	0.016	0.059	0.004	0.124	0.132
		Beta	0.087	0.012	0.389	0.309	0.326	0.333
		P value	0.433	-0.356 **0.001**	**0.001**	**0.022**	**0.005**	**0.002**
**MSA**	CSF	B	-0.002	0.001	0.13	-0.011	0.202	0.062
		95% CI	-0.006-0.002	-0.060-0.062	-0.029-0.054	-0.030-0.008	-0.062-0.466	-0.314-0.438
		SE	0.002	0.030	0.021	0.009	0.132	0.188
		Beta	-0.117	0.004	0.082	-0.327	0.186	0.040
		P value	0.347	0.976	0.551	0.234	0.131	0.744
	Plasma	B	0.002	-0.061	0.016	0.01	0.337	-0.169
		95% CI	-0.002-0.005	-0.144-0.022	-0.027-0.058	-0.017-0.019	0.014-0.660	-0.616-0.277
		SE	0.002	0.041	0.021	0.008	0.161	0.221
		Beta	0.111	-0.229	0.111	0.049	0.282	-0.119

		P value	0.430	0.144	0.461	0.885	**0.041**	0.448
**PSP/CBS**	CSF	B	0.001	0.001	0.202	0.005	-0.126	0.502
		95% CI	-0.004-0.006	-0.035-0.037	-0.091-0.494	-0.008-0.018	-1.258-1.007	-0.001-1.006
		SE	0.003	0.018	0.143	0.006	0.552	0.251
		Beta	0.060	0.008	0.245	0.198	-0.044	0.273
		P value	0.672	0.958	0.170	0.463	0.822	0.051
	Plasma	B	-0.003	-0.035	0.176	0.013		0.229
		95% CI	-0.008-0.002	-0.062--	-0.040-0.392	-0.007-0.033		-0.385-0.844
		SE	0.002	0.008	0.105	0.009	-	0.302
		Beta	-0.218	0.013	0.325	0.439		0.131
		P value	0.176	-0.407	0.105	0.177		0.453
				0.013				
**DLB**	CSF	B	<0.001	-0.006	0.025	-0.001	0.185	0.106
		95% CI	-0.001-0.002	-0.034-0.022	-0.213-0.262	-0.029-0.027	-0.261-0.632	-0.160-0.373
		SE	0.001	0.014	0.117	0.013	0.219	0.133
		Beta	0.015	-0.052	0.035	-0.014	0.148	0.101
		P value	0.907	0.693	0.835	0.952	0.404	0.428
	Plasma	B	0.001	0.004	0.005	-0.020	0.168	0.220
		95% CI	-0.002-0.005	-0.048-0.055	-0354-0.363	-0.065-0.024	-0.578-0.915	-0.273-0.714
		SE	0.002	0.025	0.171	0.020	0.352	0.242
		Beta	0.124	0.028	0.006	-0.291	0.119	0.161
		P value	0.493	0.881	0.978	0.335	0.640	0.370

List of abbreviations: PD Parkinson's disease; PDD PD dementia; DLB dementia with Lewy bodies; MSA multiple system atrophy; PSP/CBS progressive supranuclear palsy/corticobasal syndrome; LP lumbar puncture; MMSE Mini-mental state examination; UPDRS Unified Parkinson's Disease Rating Scale.

[1]Amyloid ratio evaluated as Aβ42/40 <0.65 (presence or absence).

Supplementary Table 8. Associations between NfL values and clinical parameters (adjusted for age).

**Diagnostic Group**	**NfL source**	**Coefficient**	**Time onset to LP**	**MMSE**	**Hoehn & Yahr**	**UPDRS-III**	**Orthostatic hypotension**	**Amyloid ratio[1]**
**PD/PDD**	CSF	B	0.001	-0.017	0.143	0.022	0.004	0.166
		95% CI	-0.001-0.002	-0.036-0.003	0.036-0.249	0.014-0.029	-0.175-0.182	-0.034-0.366
		SE	0.001	0.010	0.054	0.004	0.090	0.101
		Beta	0.061	-0.125	0.222	0.215	0.003	0.116
		P value	0.369	0.101	**0.009**	**0.019**	0.968	0.102
	Plasma	B	-0.001	-0.020	0.090	0.003	0.140	0.188
		95% CI	-0.002-0.001	-0.042-0.001	-0.019-0.199	-0.003-0.010	-0.079-0.359	-0.050-0.427
		SE	0.001	0.011	0.055	0.003	0.110	0.120

		Beta	-0.086	-0.183	0.174	0.116	0.126	0.150
		P value	0.363	0.062	0.105	0.303	0.207	0.119
**MSA**	CSF	B	-0.002	0.012	0.015	-0.016	0.203	0.069
		95% CI	-0.006-0.002	-0.053-0.078	-0.027-0.057	-0.038-0.006	-0.063-0.469	-0.335-0.474
		SE	0.002	0.008	0.021	0.010	0.133	0.202
		Beta	-0.117	0.056	0.099	-0.484	0.187	0.045
		P value	0.349	0.706	0.478	0.130	0.132	0.733
	Plasma	B	0.002	-0.091	0.013	-0.001	0.336	-0.213
		95% CI	-0.002-0.005	-0.182-0.000	-0.030-0.056	-0.023-0.022	0.010-0.662	-0.700-0.274
		SE	0.002	0.045	0.021	0.010	0.162	0.241
		Beta	0.114	-0.342	0.093	-0.033	0.281	-0.150
		P value	0.421	0.050	0.546	0.939	0.043	0.382
**PSP/CBS**	CSF	B	0.001	0.007	0.188	0.002	-0.257	0.442
		95% CI	-0.004-0.006	-0.030-0.044	-0.113-0.488	-0.013-0.016	-1.315-0.801	-0.065-0.950
		SE	0.003	0.018	0.147	0.007	0.515	0.253
		Beta	0.062	0.057	0.228	0.074	-0.090	0.240
		P value	0.657	0.703	0.212	0.802	0.622	0.086
	Plasma	B	-0.004	-0.024	0.181	0.008		-0.072
		95% CI	-0.008-0.001	-0.051-0.002	-0.014-0.376	-0.010-0.027		-0.603-0.459
		SE	0.002	0.013	0.094	0.008	-	0.261
		Beta	-0.234	-0.284	0.334	0.290		-0.041
		P value	0.097	0.066	0.067	0.328		0.783
**DLB**	CSF	B	<0.001	0.002	-0.043	-0.001	0.233	0.042
		95% CI	-0.002-0.001	-0.026-0.030	-0.298-0.211	-0.029-0.027	-0.213-0.679	-0.228-0.312
		SE	0.001	0.014	0.125	0.013	0.219	0.135
		Beta	-0.033	0.020	-0.061	-0.019	0.186	0.040
		P value	0.797	0.880	0.731	0.938	0.295	0.755
	Plasma	B	<0.001	0.021	-0.108	-0.018	0.231	0.108
		95% CI	-0.003-0.004	-0.026-0.069	-0.429-0.213	-0.063-0.027	-0.450-0.912	-0.358-0.574
		SE	0.002	0.023	0.153	0.020	0.319	0.228
		Beta	0.049	0.159	-0.145	-0.254	0.163	0.079
		P value	0.770	0.364	0.488	0.400	0.480	0.639

List of abbreviations: PD Parkinson's disease; PDD PD dementia; DLB dementia with Lewy bodies; MSA multiple system atrophy; PSP/CBS progressive supranuclear palsy/corticobasal syndrome; LP lumbar puncture; MMSE Mini-mental state examination; UPDRS Unified Parkinson's Disease Rating Scale.

[1]Amyloid ratio evaluated as Aβ42/40 <0.65 (presence or absence).

GUIDANCE CHECHLIST: STARD

Section & Topic	No	Item	Reported on page #
**TITLE OR ABSTRACT**			
	**1**	Identification as a study of diagnostic accuracy using at least one measure of accuracy<br>(such as sensitivity, specificity, predictive values, or AUC)	1
**ABSTRACT**			
	**2**	Structured summary of study design, methods, results, and conclusions<br>(for specific guidance, see STARD for Abstracts)	2
**INTRODUCTION**			
	**3**	Scientific and clinical background, including the intended use and clinical role of the index test	3
	**4**	Study objectives and hypotheses	3
**METHODS**			
Study design	**5**	Whether data collection was planned before the index test and reference standard<br>were performed (prospective study) or after (retrospective study)	4
Participants	**6**	Eligibility criteria	4
	**7**	On what basis potentially eligible participants were identified<br>(such as symptoms, results from previous tests, inclusion in registry)	4-5
	**8**	Where and when potentially eligible participants were identified<br>(setting, location and dates)	4
	**9**	Whether participants formed a consecutive, random or convenience series	4
Test methods	**10a**	Index test, in sufficient detail to allow replication	5-6
	**10b**	Reference standard, in sufficient detail to allow replication	5
	**11**	Rationale for choosing the reference standard (if alternatives exist)	3-5
	**12a**	Definition of and rationale for test positivity cut-offs or result categories<br>of the index test, distinguishing pre-specified from exploratory	5-6

	**12b**	Definition of and rationale for test positivity cut-offs or result categories<br>of the reference standard, distinguishing pre-specified from exploratory	5-6
	**13a**	Whether clinical information and reference standard results were available<br>to the performers/readers of the index test	6
	**13b**	Whether clinical information and index test results were available<br>to the assessors of the reference standard	NA
Analysis	**14**	Methods for estimating or comparing measures of diagnostic accuracy	6
	**15**	How indeterminate index test or reference standard results were handled	5,6
	**16**	How missing data on the index test and reference standard were handled	NA
	**17**	Any analyses of variability in diagnostic accuracy, distinguishing pre-specified from exploratory	NA
	**18**	Intended sample size and how it was determined	NA
**RESULTS**			
Participants	**19**	Flow of participants, using a diagram	NA
	**20**	Baseline demographic and clinical characteristics of participants	7
	**21a**	Distribution of severity of disease in those with the target condition	7 (Table 1)
	**21b**	Distribution of alternative diagnoses in those without the target condition	NA
	**22**	Time interval and any clinical interventions between index test and reference standard	7 (Table 1)
Test results	**23**	Cross tabulation of the index test results (or their distribution)<br>by the results of the reference standard	10 (Table 3)
	**24**	Estimates of diagnostic accuracy and their precision (such as 95% confidence intervals)	10
	**25**	Any adverse events from performing the index test or the reference standard	NA
**DISCUSSION**			
	**26**	Study limitations, including sources of potential bias, statistical uncertainty, and generalisability	15

	**27**	Implications for practice, including the intended use and clinical role of the index test	15
**OTHER INFORMATION**			
	**28**	Registration number and name of registry	NA
	**29**	Where the full study protocol can be accessed	NA
	**30**	Sources of funding and other support; role of funders	16
