# Supplementary material for: Neurofilament light chain and α-synuclein RT-QuIC as differential diagnostic biomarkers in parkinsonisms and related syndromes
Source: NPJ Parkinsons Dis. 2021 Oct 11;7:93. doi: 10.1038/s41531-021-00232-4 (PMC8505434; doi:10.1038/s41531-021-00232-4)
Supplement: Supplementary file 2 — Reporting summary [file 41531_2021_232_MOESM2_ESM.pdf]

nature research

Corresponding author(s): Prof. Piero Parchi

Last updated by author(s): Jun 2, 2021

Reporting Summary

Nature Research wishes to improve the reproducibility of the work that we publish. This form provides structure for consistency and transparency in reporting. For further information on Nature Research policies, see our Editorial Policies and the Editorial Policy Checklist.

Statistics

For all statistical analyses, confirm that the following items are present in the figure legend, table legend, main text, or Methods section.

n/a	Confirmed	
☐	☒	The exact sample size (*n*) for each experimental group/condition, given as a discrete number and unit of measurement
☐	☒	A statement on whether measurements were taken from distinct samples or whether the same sample was measured repeatedly
☐	☒	The statistical test(s) used AND whether they are one- or two-sided<br>*Only common tests should be described solely by name; describe more complex techniques in the Methods section.*
☐	☒	A description of all covariates tested
☐	☒	A description of any assumptions or corrections, such as tests of normality and adjustment for multiple comparisons
☐	☒	A full description of the statistical parameters including central tendency (e.g. means) or other basic estimates (e.g. regression coefficient) AND variation (e.g. standard deviation) or associated estimates of uncertainty (e.g. confidence intervals)
☐	☒	For null hypothesis testing, the test statistic (e.g. *F*, *t*, *r*) with confidence intervals, effect sizes, degrees of freedom and *P* value noted<br>*Give P values as exact values whenever suitable.*
☒	☐	For Bayesian analysis, information on the choice of priors and Markov chain Monte Carlo settings
☒	☐	For hierarchical and complex designs, identification of the appropriate level for tests and full reporting of outcomes
☐	☒	Estimates of effect sizes (e.g. Cohen's *d*, Pearson's *r*), indicating how they were calculated

Our web collection on statistics for biologists contains articles on many of the points above.

Software and code

Policy information about availability of computer code

Data collection	File Maker Pro 16 Advanced version 16.0.2.205 (Claris International Inc., USA)
Data analysis	IBM SPSS Statistics version 24 (IBM, Armonk, NY, USA) and GraphPad Prism 8 (GraphPad Software, La Jolla, CA, USA) software were used for statistical analysis of data.

For manuscripts utilizing custom algorithms or software that are central to the research but not yet described in published literature, software must be made available to editors and reviewers. We strongly encourage code deposition in a community repository (e.g. GitHub). See the Nature Research guidelines for submitting code & software for further information.

Data

Policy information about availability of data

All manuscripts must include a data availability statement. This statement should provide the following information, where applicable:

- Accession codes, unique identifiers, or web links for publicly available datasets
- A list of figures that have associated raw data
- A description of any restrictions on data availability

The data that support the findings of this study are available from the corresponding author upon reasonable request.

Field-specific reporting

Please select the one below that is the best fit for your research. If you are not sure, read the appropriate sections before making your selection.

☒ Life sciences ☐ Behavioural & social sciences ☐ Ecological, evolutionary & environmental sciences

For a reference copy of the document with all sections, see nature.com/documents/nr-reporting-summary-flat.pdf

Life sciences study design

All studies must disclose on these points even when the disclosure is negative.

Sample size	Participants were recruited between 2007 and 2019 in a consecutive series. No sample-size calculation was performed, all available samples fulfilling selection criteria were used.
Data exclusions	No data were excluded from the analysis.
Replication	The reproducibility of biomarkers quantification in biofluid was evaluated using mean intra- and inter-assay coefficients of variation (CVs). The mean intra- and inter-assay CVs for plasma NfL were 4% and 11%. The inter-assays CVs for CSF total-tau, phospho-tau, amyloid-beta1-42, and amyloid-beta1-40 were <8% for all biomarkers. The mean intra- and inter-assays CVs for CSF NfL were 2% and 10%.<br>The reproducibility of the RT-QuIC assay in our laboratory was demonstrated in Rossi, M. et al., Acta Neuropathol. 2020;140:49-62.
Randomization	The analysed cohort included 355 patients affected by parkinsonism of probable neurodegenerative etiology, 49 patients affected by syndromes that may precede the onset of parkinsonism (i.e. isolated autonomic failure and REM-Sleep Behaviour disorder) and 72 non-neurodegenerative controls. We selected cases with a probable clinical diagnosis at last follow-up and available plasma and/or CSF samples. Samples were allocated into experimental groups based on the probable clinical diagnosis of patients.<br>Relevant covariates are age and gender. Age was associated with plasma and CSF NfL levels, whereas gender showed no effect on blood and CSF biomarker values. Accordingly, we adjusted for age for all comparisons of plasma and CSF NfL between groups.
Blinding	Investigators were blinded to group allocation during data analysis.

Reporting for specific materials, systems and methods

We require information from authors about some types of materials, experimental systems and methods used in many studies. Here, indicate whether each material, system or method listed is relevant to your study. If you are not sure if a list item applies to your research, read the appropriate section before selecting a response.

Materials & experimental systems

n/a	Involved in the study	
☒	☐	Antibodies
☒	☐	Eukaryotic cell lines
☒	☐	Palaeontology and archaeology
☒	☐	Animals and other organisms
☐	☒	Human research participants
☒	☐	Clinical data
☒	☐	Dual use research of concern

Methods

n/a	Involved in the study	
☒	☐	ChIP-seq
☒	☐	Flow cytometry
☒	☐	MRI-based neuroimaging

Human research participants

Policy information about studies involving human research participants

Population characteristics	Age and gender are the covariate-relevant characteristics of the populations analysed. Age was associated with plasma NfL levels and with CSF NfL levels, whereas gender showed no effect on blood and CSF biomarker values.
Recruitment	Participants were recruited based on their probable diagnosis at last follow-up made by a movement disorder specialist at the Institute of Neurological Sciences of Bologna (ISNB) between 2007 and 2019, and on plasma and/or CSF samples availability. 61 patients belonged to the BOPROPARK cohort.<br>We used a clinical criteria and not neuropathological data for the study participants' diagnoses. However, medical doctors specialising in movement disorders established the clinical diagnoses in patients followed over time with reassessments at each follow-up visit. Moreover, we included only patients fulfilling the criteria for probable or clinically established disease. The use of a unique set of selection criteria for all patients should grant the absence of impact on results.
Ethics oversight	The study was approved by the ethics committee “Area Vasta Emilia Centro” (approval numbers AVEC: 09070, 17093, 18025, and 18027).

Note that full information on the approval of the study protocol must also be provided in the manuscript.
